# Self‐Assembly Engineering of Fullerene‐Like Polyhedra: V_60_, V_66_, V_72_ From {MV_5_} Pentagonal Second Building Block

**DOI:** 10.1002/advs.202408863

**Published:** 2025-03-24

**Authors:** Hongmei Gan, Linyan Bao, Na Xu, Baoshan Hou, Xinlong Wang, Zhongmin Su

**Affiliations:** ^1^ State Key Laboratory of Supramolecular Structure and Materials Jilin University Changchun Jilin 130024 China; ^2^ Key Laboratory of Chemistry and Chemical Engineering on Heavy‐Carbon Resources Yili Normal University Yining Xinjiang 835000 China; ^3^ Key Lab of Polyoxometalate Science of Ministry of Education Northeast Normal University Changchun Jilin 130024 China

**Keywords:** fullerene‐like cages, metal–organic polyhedra, supramolecular self‐assembly

## Abstract

The synthesis of hollow fullerene‐like cages has always been an attractive goal for researchers. Nevertheless, such molecular design blueprints are often hampered by the unmet customization of assembly strategy and suitable five‐membered ring molecular tiles for building spheres. Herein, a novel spherical molecular cage V_60_‐MOP is successfully built by connecting 12 homo‐metallic pentagonal {V_5_S} tiles with benzene‐1,3,5‐tricarboxylate (BTC) ligands through coordination‐driven self‐assembly and exhibits a similar geometry to the C_60_ molecule. As far as is known, this {V_5_S} cluster with the shape of a hollow pentagram is entirely new and is first discovered in metal–organic polyhetra (MOPs). The other two pentagonal motifs are also customized by inserting [VO_5_] or [MoO_5_] in the center of {V_5_S} through in situ modification, which also led to another isomorphic V_72_‐MOP and V_60_Mo_12_‐MOP fullerene‐like cages. The three spherical cages reported above all exhibit regular icosahedral symmetry. Notably, a fascinating structure V_66_‐MOP is constructed from two different pentagonal motifs {V_5_S} and {VV_5_S}, with reduced symmetry, although the molecule remains a sphere in appearance, which is rare in fullerene‐shaped structures. In addition, the series of fullerene‐like V‐MOPs are active in the oxidation of sulfides to produce sulfoxides or sulfones with high conversion.

## Graphical abstract

A precise self‐assembly of fullerene‐like V_60_‐MOP constructed from the first discovered polyoxovanadates five‐membered ring V_5_ units is discovered. In situ modifications are successfully used to insert {MO_5_} (M = V/Mo) in the center of the V*_5_* ring, resulting in three other isomorphic spherical cages V_66_‐MOP, V_72_‐MOP, and V_60_Mo_12_‐MOP. The discovered V*_5_* unit provides an important active site in catalytic reactions.

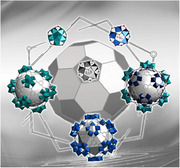

## 1 Introduction

Giant supramolecular clusters are visually attractive due to their intriguing architecture comparable to biological macromolecules and the ordered oxides can offer a platform to precisely interrogate the relationships between structures and activity.^[^ ^1^ ^]^ Polyoxometalates (POMs) can be hailed as the ideal molecular prototypes for exploring and mimicking larger and more complex nanoclusters,^[^ ^2^, ^3^, ^4^, ^5^ ^]^ because they combine geometrical or chemical tunability with classic functionalities of metal–oxide to provide excellent nano‐systems that can be exploited for prompt or potential applications in the fields of catalysis, energy storage, biology and biomedicine.^[^ ^6^, ^7^, ^8^, ^9^, ^10^ ^]^ Chemists have been exploring challenging synthetic pathways to build a variety of large POMs‐based motifs. As early as the beginning of the 21st century, Müller's group reported the most prominent example of POMs cluster Mo_368_ “hedgehog,” which is also the largest inorganic metal cluster that has not been surpassed so far.^[^ ^2^ ^]^ In purely inorganic assemble, giant Mo clusters usually appear as hollow or toroidal capsule entities,^[^ ^3^, ^11^, ^12^, ^13^ ^]^ while high‐nuclearity clusters of other sub‐type POMs are rarely observed due to the condensation, instinctively tending to form low‐nuclearity aggregate. Particularly, the development of giant polyoxovanadates (POVs)‐clusters chemistry has long lagged.

Coordination‐driven self‐assembly has established a powerful pathway for linking POMs building blocks with other metal ions or organic ligands to aggregate into giant clusters. However, this strategy is most commonly used to construct cluster‐of‐cluster aggregates for polyoxotungstates or polyoxoniobates.^[^ ^14^, ^15^, ^16^, ^17^, ^18^ ^]^ Significant advantages have been observed in POVs that metal clusters are more conducive to modular pre‐organization than single metal ions, and V ions are able to efficiently polarize the terminal O^2−^ ligands to provide directional “bowl” subunits which are important for the formation of “closed” highly symmetrical capsules.^[^ ^19^, ^20^, ^21^, ^22^ ^]^ In addition, the short V─O bonds with dπ‐pπ contributions further increase the dynamic stability of this kind of subunit. Dinuclear and tetranuclear secondary building units (SBUs) have been controlled to assemble into various high‐nuclearity hybrid cluster cages under the induction of anion templates by Schmitt and colleagues.^[^ ^23^, ^24^ ^]^ However, further expansion of structures has also encountered a bottleneck by this approach, in which the connection between metal units and a single functional group is relatively random, and it instinctively tends to form small entities without cavities. Rigid dual‐ or multi‐connected organic isolators proved to be more suitable for constructing highly symmetric cages, and several high‐nuclearity aggregates with tetrahedral,^[^ ^25^, ^26^, ^27^ ^]^ octahedral, and cubic topologies have been obtained by the controlled assembly.^[^ ^25^, ^28^, ^29^, ^30^ ^]^ These POVs‐motifs can provide well‐defined accessible sites for organic ligands, and offer great possibilities for predicting self‐assembled structures by the Reticular Chemistry Structure Resource (RCSR), i.e., 3‐connected (3‐c) and 4‐c SBUs can be oriented to build metal‐organic polyhetra (MOPs) with tetrahedral and octahedral symmetry, respectively.^[^ ^31^ ^]^ Nevertheless, giant spherical structures with high symmetry have rarely been reported even in POMs chemistry, and the representative examples include {Mo_102_},^[^ ^32^ ^]^ {Mo_132_},^[^ ^33^ ^]^ {W_72_V_30_}^[^ ^34^ ^]^ and the latest {V_30_Nb_12_}.^[^ ^35^ ^]^ The key challenges are the lack of adapted building parts and assembly rules, which are far from the precise and efficient pre‐organized synthesis for bottom‐up self‐assembly in analogous nature.

**Figure 1:**
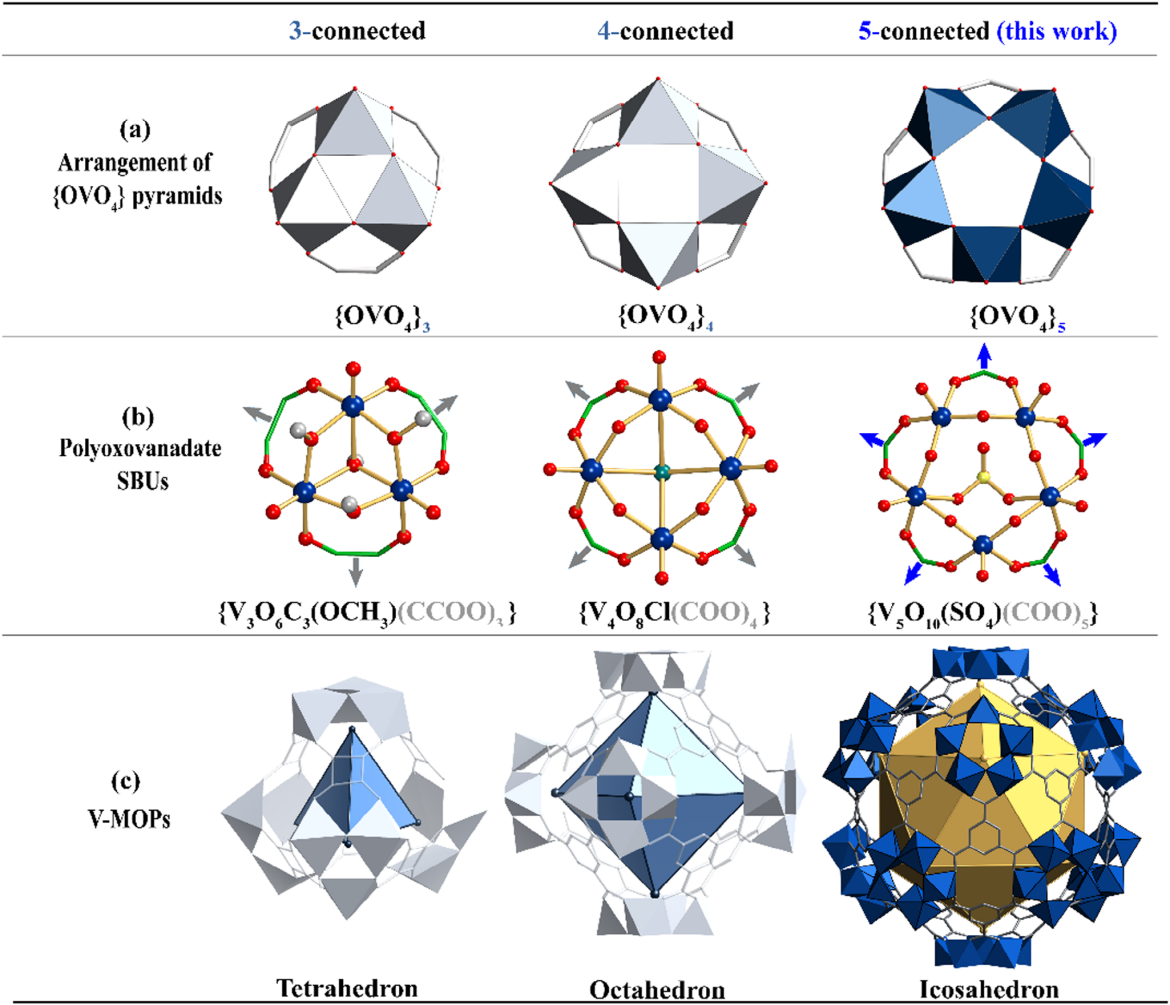
a) Polyhedral structures of **V*_n_*** SBUs ({OVO_4_}_n_): **V_3_**, **V_4_**, **V_5_**. b) Ball‐and‐stick views of **V*_n_***‐SBUs. c) Structures and polyhedral topology of V‐MOPs.

The geometry of the spherical fullerene exhibits icosahedral symmetry, which is also the highest possible symmetry for a molecule. To construct an icosahedral structure following the RCSR framework, 12 5‐coordinated vertices and twenty rigid faces are typically required. In addition, the arrangement of 12 pentagons is crucial for the generation of spherical structures due to the inability of the pentagon to form dense planar stacks. However icosahedral coordination molecular cages (also called MOPs) have rarely been reported.^[^ ^36^, ^37^, ^38^, ^39^ ^]^ Therefore, developing adapted pentagonal SBUs is the main challenge to modularly organize a fullerene‐like spherical structure. A circular hollow **V*_n_*** (The value of *n* corresponds to the final connection numbers of the SBUs) have been found in VMOPs, whose structure is characterized by arrangement of {OVO_4_} square pyramids by sharing the vertices on the same side of the bottom square surface (**Figure** **1**a), including two units **V*_3_***: {V_3_O_6_C_3_(OCH_3_)} and **V*_4_***: {V_4_O_8_C} (Figure 1b).^[^ ^20^, ^25^ ^]^ Topological analysis shows that **V*_3_*** and **V*_4_*** can be oriented to construct tetrahedra and octahedra respectively (Figure 1c). Next, **V*_5_*** of the same class will be a promising building block for assembling an icosahedron. In this study, the common rigid tricarboxylic acid ligands BTC (BTC = benzenetricarboxylic acid) are selected as the smallest triangle molecular tiles that participated in the self‐assembly process. As expected, we have obtained a supramolecular cage **V_60_‐MOP**: (H_2_NMe_2_)_84_ {[V_5_O_10_(SO_4_)]_12_(btc)_20_}·(solvent), which is composed of 12 hollow **V*_5_*** SBUs:{V_5_O_10_SO_4_} bridged by 20 organic BTC spacers, and 60 vanadium atoms exhibit the same arrangement as C_60_. With further in situ modification, another two isomorphic cages **V_72_‐MOP** and **V_60_Mo_12_‐MOP** have also been successfully assembled. Notably, an unusual intermediate **V_66_‐MOP** was unanticipatedly crystallized in this complex self‐assembly system. As far as we know, they are the three highest nuclear vanadium clusters.

## 2 Results and Discussion

Experience has shown that different anions can induce and stabilize different vanadium‐based SBUs, Cl^−^ or Br^−^ can control the formation of {V_4_O_8_} or {VV_4_O_9_} units, and the presence of H_2_O has led to the formation of dinuclear groups. We choose VOSO_4_ as the vanadium source providing SO_4_^2−^ anions to induce the expected novel SBUs. At first, no matter how we changed the reaction conditions, including temperature, molar feed ratios, and solvent ratios, only tetrahedral tetramers (**VMOP‐14**) consisted of {V_6_O_6_(OCH_3_)_9_(SO_4_)} units could be obtained with the participation of only VOSO_4_ and BTC.^[^ ^27^ ^]^ This may be because a larger number of small cages in the self‐assembly process leads to a smaller entropy, but a fact has been proved that SO_4_^2−^ can be used as an anion template to stabilize advanced ringlike SBUs. Until the steam‐thermal method is adopted with mixed DMA/CH_3_OH solvents (1/2,v/v), brown octahedral crystals of **V_60_‐MOP** with the formula {(H_2_NMe_2_)_84_{[V_5_O_10_(SO_4_)]_12_(btc)_20_}·(solvent) were obtained by keeping it at 130 °C for two days. (Supporting Information for detailed synthesis methods). Compared with a traditional solvothermal method, this method can significantly slow down the nucleation rate through the slow contact between the solvent and the solid.

**Figure 2:**
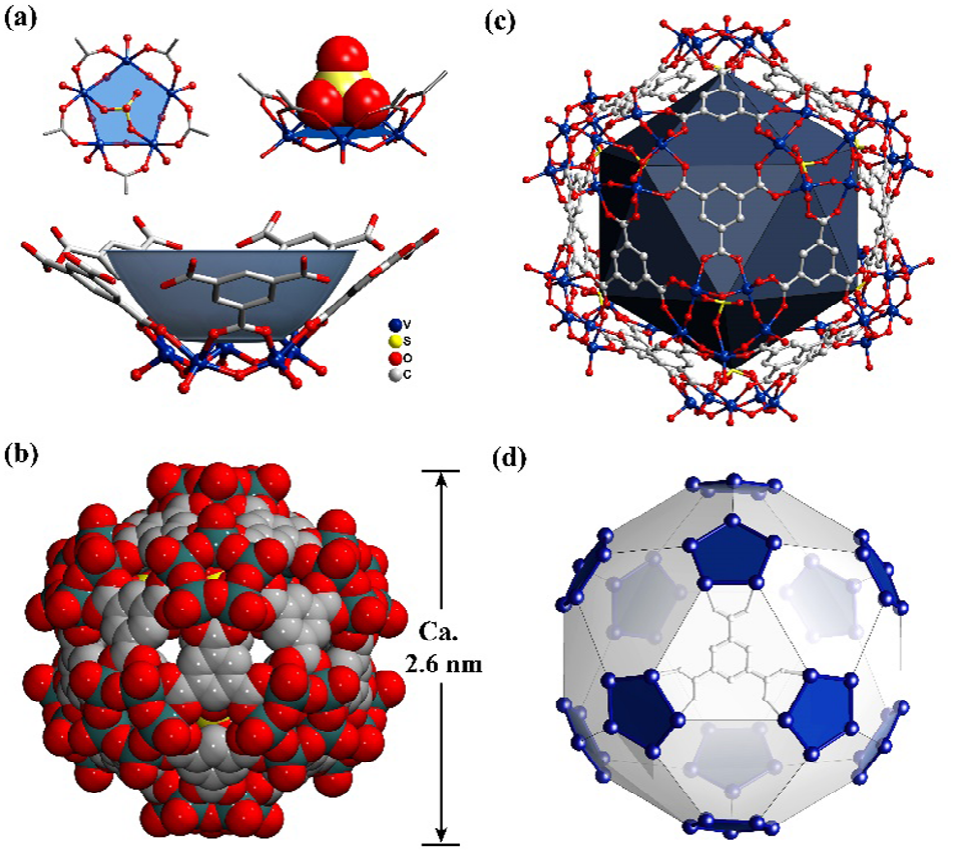
a) Ball‐and‐stick views of bowl‐shaped **V*_5_*** SBU: {V_5_O_10_(SO_4_)}. b) space‐filling views of the diameter of the **V_60_‐MOP** cage. c) Ball‐and‐stick views of **V_60_‐MOP,** nearly spherical structure with icosadral symmetry. d) V atom‐based skeleton of **V_60_‐MOP in** ball‐and‐stick representation.

Single‐crystal X‐ray diffraction analysis shows that the **V_60_** crystallizes in the cubic system with space group *Fm*−3 and consists of 60 vanadium atoms and 20 completely deprotonated BTC ligands. Each group of five vanadium atoms forms a cyclic {V_5_O_10_(SO_4_)} unit that can be bridged by five carboxyl groups leading to a bowl‐shaped **V*_5_*** SBU, which is stabilized by a sulfate located at its focal point (**Figure** **2**a). The five vanadiums are distributed almost in the same plane with a distance between V···V range from 3.368–3.405Å, and the five‐membered ring is formed by the μ_2_‐O hand in hand bridge. These vanadium ions can be divided into two coordination modes (Figure S1, Supporting Information): i) one vanadium atom is coordinated with five oxygen atoms to form a tetragonal pyramidal {VO_5_} cluster in which the V─O bonds distances range from 1.575 to 2.078 Å; ii) the other four vanadium atoms form a six‐coordinate octahedral {VO_6_} clusters, and V─O bonds distances are between 1.582 and 2.416 Å. Two {VO_6_} clusters are separated by a {VO_5_}. In this arrangement the O–V–O angle range is 70.9–163.5°, and the V–O–V angle range is 133.9–138.0°. Bond valence sum (BVS) calculations (Table S2, Supporting Information) reveal that all V ions show +4 valence, which is consistent with the X‐ray photoelectron spectroscopy (XPS) test results (Figure S2, Supporting Information). Ultimately, the twelve pentanuclear ring motifs are connected by 3‐connected organic spacers, uniformly arranged into a spherical cage with a diameter of 2.6 nm, and the overall skeleton includes 60 vanadium, 180 carbon, 288 oxygen, and 12 sulfur atoms (Figure 2b).

An icosahedron can be directly built to improve our understanding of the geometry by connecting the 12 sulfur atoms located at the focal point of the **V_5_** units inside the cage (Figure 2c). This icosahedron contains two kinds of almost equal length edges (10.51 Å green, 10.58 Å yellow), forming 12 isosceles triangle faces and 8 congruent triangle faces exactly parallel to the BTC ligand tiles separated by 2.77 Å. The triangular interior angles vary from 59.56–60.22°, and the dihedral angles vary from 137.7–138.2°, which are mostly close to the dihedral angle of the ideal icosahedron 138.3° (Figure S3, Supporting Information). In view of the above analysis, **V_60_‐MOP** can be assigned to an approximated *I_h_* point group with highly symmetric icosahedral symmetry. Remarkably, the spatial arrangement of the 60 vanadium atoms coincides with fullerene C_60_ and forms a truncated icosahedron (Figure 2d). Following the basic minimal transitivity (simplest possible) principle of reticular chemistry, the simplest topology of **V_60_‐MOP** will consist of two types of vertices, including twelve 5‐c SBUs and twenty 3‐c organic nodes. Then, a Rhombic triacontahedron polyhedron is obtained, and its RCSR code is **trc** (Figure S4, Supporting Information). Moreover, the packing arrangement shows that one cage is located in the center of the cubic, and the other eight cages are seated as the vertices of the cubic surrounding the central cage (Figure S5, Supporting Information). When we treat the icosahedron as an 8‐c node, **V_60_‐MOP** enables it to be reduced into a classical ***bcu*** topology.

**Figure 3:**
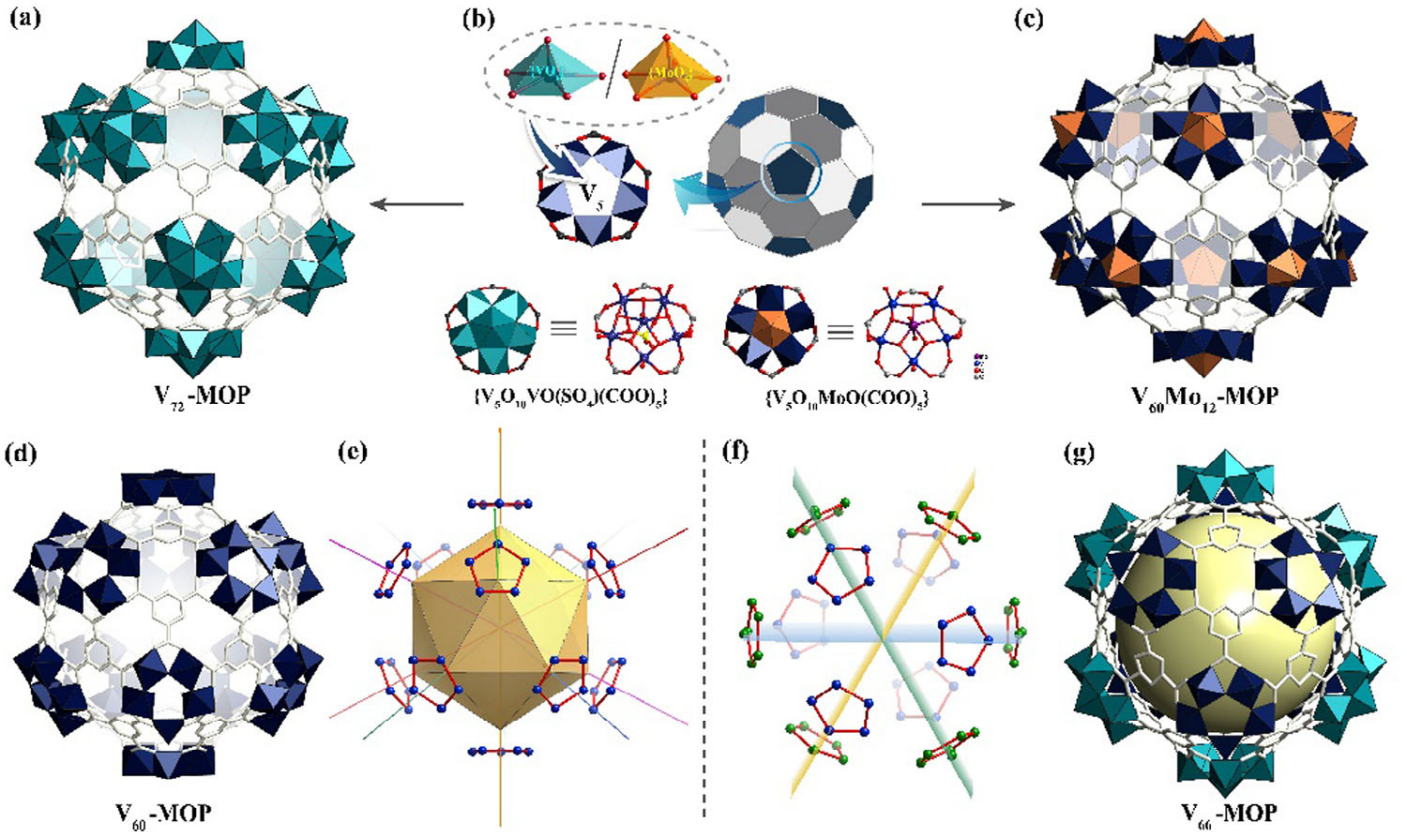
The overall views of the structures of V‐MOPs of **V_72_‐MOP** a), **V_60_Mo_12_‐MOP** c), **V_60_‐MOP** d), **V_66_‐MOP** g). V‐based **5‐c** SBUs are presented by polyhedra and BTC ligands are shown by sticks. b) the formation of {MoV_5_O_11_(SO_4_)} and {VV_5_O_11_(SO_4_)} SBUs by introducing {MO_6_}, polyhedral presentation of fullerene‐like **V_60_‐MOP**. e,f) Molecular symmetry‐operation of molecules for **V_60_‐MOP** and **V_66_‐MOP**.

Obviously, the **V_5_** unit of pentagons is the key to guiding the assembly of fullerene‐like spherical structures. In order to obtain more applicable SBUs with C*_5_* symmetry for the construction of icosahedra and inspired by the classical {(Mo)Mo_5_},^35^ we would like to modify the **V_5_** SBUs in situ without changing the overall icosahedral structure. The center of the **V_5_** ring is just enough to insert a {MO_6_} pentagonal cone through the edge‐sharing, and such a local modification is analogous to the same relationship between the reported {V_4_Cl} and {V(V_4_Cl)}‐SBUs, which have been used to construct octahedra.^26^ After unremitting efforts, the 6‐coordinated {VO_6_} or {MoO_6_} were successfully inserted into the center and respectively yielded two new icosahedral cages **V_72_‐MOP** and **V_60_Mo_12_‐MOP** (**Figure** **3**).

Single‐crystal structural analyses show that the **V_72_‐MOP** crystallizes in the triangular space group *R*‐3*m* with a = 29.84, b = 29.84, c = 79.52. Compared to **V_60_‐MOP**, the whole skeleton of **V_72_‐MOP** still maintains the original topology of **trc** with icosahedral symmetry but a slightly larger external dimension than that of **V_60_‐MOP** at ≈29.4 Å (the furthest distance between two opposing oxygen atoms). Meanwhile, a new hexanuclear *C*_5_ symmetry SBU {VV_5_O_11_(SO_4_)} appeared, with a pentagonal pyramid {VO_6_} at the center sharing the bottom edges with five {VO_5_} tetragonal cones around it (Figure 3b). To the best of our knowledge, it is the highest‐nuclearity POV cage to date, containing 72 vanadium ions. Using the same method of in situ modification, another new structure **V_60_Mo_12_‐MOP** was likewise obtained by the introduction of molybdates. The center vacancy of {V_5_O_10_(SO4)} units was successfully inserted by 5‐coordinated {MoO_6_} pyramids, giving a new {MoV_5_O_11_(SO_4_)} SBU, which was then linked with BTC to finally obtain **V_60_Mo_12_‐MOP** isomorphic to **V_72_‐MOP** (Figure 3c). It crystallizes in the *R‐*3*m* space group with an anionic composition of [(MoV_5_O_11_SO_4_)_12_(BTC)_20_]^12−^. Like the other two fullerene‐like cages, **V_60_Mo_12_‐MOP** contains a solvent‐accessible approximately spherical cavity with a volume of ≈3997.13 Å^3^(Figure S6, Supporting Information).

In view of the high similarity between the **V_60_‐MOP** and **V_72_‐MOP** structures, this prompted us to think about the self‐assembly process of this saturated 72‐core icosahedron. **V_72_** appears to be the final product of continued step‐by‐step assembly through precise positioning at the atomic level on the base of **V_60_**. It is easy to wonder whether a stepwise reaction could achieve such a structural modification of **V_60_**, but when we tried to continue the reaction with the already obtained **V_60_‐MOP** as a precursor, supplemented with V or Mo, we were not able to obtain any new crystals. The results suggest that the self‐assembly process of such a 72‐core icosahedron should probably be a thermodynamically controlled one‐step assembly of stabilized SBUs in solution, and also responds to the modular assembly property of POV‐based MOPs, in which the self‐assembly process starts with the formation of stable 5‐connected SBUs, followed by the formation of icosahedral structures through a directed link with 3‐connected organic ligands.

Unexpectedly, another novel structure **V_66_‐MOP** was obtained when we increased the reaction temperature. Single‐crystal X‐ray diffraction analysis shows that two different kinds of 5‐c SBUs appear in the structure at the same time, and six hollow ring‐shaped {V_5_O_10_(SO_4_)} units are located in the equatorial ring of the sphere, while the other six {VV_5_O_11_(SO_4_)} units evenly distributed on both sides of the equatorial plane (Figure 3g). With such an arrangement, the symmetry of **V_66_‐MOP** no longer belongs to I*_h_* point group, which contains five *C*_5_ rotation axes, but only has three symmetry planes (Figure 3e,f). V ions in the center of the six {VV_5_O_11_(SO_4_)} units are + 5 valent, and the others are +4 valent (Table S3, Supporting Information). To assess the redox behavior, the scan‐rate‐dependent cyclic voltammogram (CV) of V_66_ was recorded (Figure S7, Supporting Information). In the positive scan, two peaks at 0.55 and 0.75 V are assigned to the oxidation of V^IV^ to V^V^, while three reduction peaks are observed at 0.75, 0.45, and 0.15 V in the reverse scan, indicating that the redox process is reversible. As the scan rate increases, the oxidation and reduction peaks shift to more positive and negative potentials, respectively. The electrospray ionization MS (ESI‐MS) and traveling wave ion mobility MS (TWIM‐MS) spectra tested in mixed solutions of DMSO and H_2_O show that **V_66_**‐MOP has good solution stability in solvents (Figure S8, Supporting Information). X‐ray powder diffraction in different solvents proves its crystal stability (Figure S9, Supporting Information). The compounds were fully characterized by X‐ray powder diffraction (Figure S10, Supporting Information), infrared spectroscopy (Figure S11 and Table S4, Supporting Information), and thermogravimetry (Figure S12, Supporting Information).

**Figure 4:**
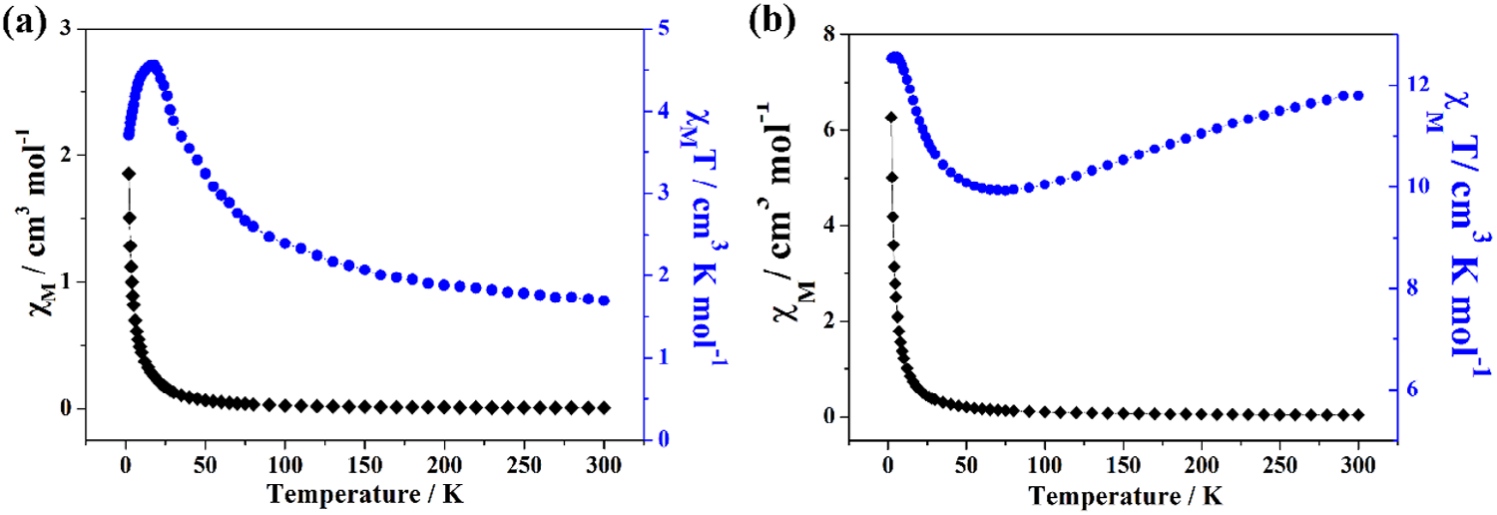
Magnetic properties of **V_60_‐MOP** a), and **V_66_‐MOP** b): *χ*_M_T as a function of temperature at 0.1 T.

Considering the structural differences present in these cages, molecular magnetic properties were further explored. **V_60_‐MOP** and **V_66_‐MOP** molecules with better yields and purity and with two different symmetry systems were selected and tested for temperature‐dependent magnetic susceptibility from 2–300 K under a magnetic field of 1 kOe. Experimental data shows the room temperature *χ*_M_T value of 1.69 and 11.79 cm^3^ K mol^−1^ corresponding to **V_60_‐MOP** and **V_66_‐MOP** separately, both of which are distinctly below the expected value of 22.5 cm^3^ K mol^−1^ for 60 noninteracting spin‐1/2 V^4+^ centers. The *χ*_M_T value of **V_60_** increases continuously along with the decreasing temperature and reaches its maximum value of 4.55 cm^3^ K mol^−1^ at 18 K. Moreover, the increasing trend with decreasing temperature at higher temperatures indicates intramolecular ferromagnetic interactions exchange between neighboring metal ions (**Figure** **4**a). For **V_66_‐MOP**, the experimental curves show a different trend. Upon cooling, *χ*_M_T value continuously decreases and reaches its minimum value of 9.93 cm^3^ K mol^−1^ at 70 K. Further lowering of the temperature gradually brought the value back up again, reaching 12.56 cm^3^ K mol^−1^ when the temperature reduces to 2 K. The plot of 1/*χ*_M_ versus T between 100–300 K obeys the Curie‐Weiss law (1/*χ*_M_ = (T − θ)/C) with C = 12.72 cm^3^ mol and a large negative θ value of −30.76 K (Figure S13, Supporting Information). Both the curve and negative θ indicate dominant antiferromagnetic exchange interactions within this cage (Figure 4b). Although the molecular magnetism of both cages is contributed by the 60 V^4+^ ion on the 12‐group five‐membered ring in the same position, the differences in magnetic properties indicate the different magnetic behavior exhibited by the V^4+^ centers in different symmetry groups. In combination with BVS, the connectivity of all V^IV^ spin centers can be represented by a spin topology that consists of 12 spin pentagons {V_5_}, where the magnetic exchange primarily is mediated by the μ_3_‐O sites, and these 12 motifs are then connected via BTC. However, the {VO_6_} unit at the center of {V_5_} can also act as a magnetic exchange pathway in V_66_, furthermore complicating the exchange connectivity. This may be the main reason for the differences in the magnetic properties.^[^ ^40^, ^41^ ^]^

Given the widespread interest in POM as an environmentally friendly redox catalyst, the catalytic oxidation of sulfides was investigated to explore the catalytic activity of V‐MOPs. Firstly, methyl phenyl sulfide (MBT) was chosen as the substrate and H_2_O_2_ as the oxidant, and the catalytic activity of several VMOPs for the reaction with methanol as solvent at room temperature was investigated. The results show that the three V‐MOPs catalysts exhibited similar catalytic activity and the substrates were almost completely converted after 1 h in all three reactions (Figure S14, Supporting Information). In contrast, the conversion rate without the presence of catalysts was only 26.46%. Furthermore, a set of parallel reactions showed little increase in conversion after the removal of the catalyst by filtration during the reaction, suggesting that the V‐MOPs compounds play a crucial role in the catalytic reaction. Next, the catalytic oxidation reactions of the other five sulfide substrates were investigated under the same reaction conditions using **V_66_‐MOP** with high yield and better phase purity. As shown in **Table** **1**, the conversion rate of diethyl sulfide reached 100% after 1 h, and the other substrates containing benzene rings, ethyl phenyl sulfide, 4‐methoxy‐methyl phenyl sulfide, and 4‐nitro‐methyl phenyl sulfide, were 99%, 99%, 78%. In this case, due to the electron‐withdrawing property of the para‐nitro group of the benzene ring, 4‐nitro‐methyl phenyl sulfide is more difficult to convert. In addition, diphenyl sulfide also can be oxidized efficiently with a conversion rate of 99% after 2 h. The dibenzothiophen cannot be complete despite an extended reaction time of up to 4 h, only with a conversion rate of 86%. After five reaction cycles, the conversion of MBT decreased by less than 5% (Figure S15, Supporting Information). Although the conversion of sulfide oxidation is considerable with **V_66_‐MOP** compounds as catalysts, a large portion of sulfide substrates is converted to the sulfoxide product, and a small portion is converted to sulfone.

**Table 1:** Different sulfides substrates oxidation reactions catalyzed by **V_66_‐MOP** under the conditions.

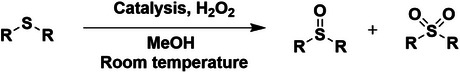				
Entry^a)^	Substrate	Time [h]	Conv. [%]^b)^	Sele. [%]^c)^
1	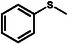	1	≥99％	98.4％
2	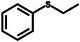	1	≥99％	96.5％
3	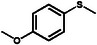	1	≥99％	98.7％
4	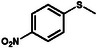	1	78.1％	95.8％
5	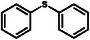	2	≥99％	90.2％
6	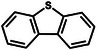	4	86.1％	89.3％

^a)^ Reaction conditions: substrate (0.4 mmol), **V_66_‐MOP** (0.002 mmol), H_2_O_2_ (1mmol), and MeOH (5 mL), Room temperature;

^b)^ Isolated conversions were calculated by GC‐MS;

^c)^ Selectivity to the corresponding sulfoxide. Selectivity to the corresponding sulfoxide determined by GC‐MS.

In order to further confirm the active sites of V‐MOPs, controlled experiments were carried out for the catalytic oxidation of MBT using VOSO_4_, and H_3_BTC as catalysts. As shown in Figure S14b (Supporting Information), the organic ligands had almost no effect on the catalytic reaction compared to the blank test, while VOSO_4_ showed significant catalytic activity with a conversion rate as high as 53%, which indicated that the vanadium clusters provide the main catalytically active sites in this system. However, the three VMOPs exhibited similar catalytic activities despite being composed of two different vanadium clusters based on SBUs. In order to further accurately identify the effects of differences in the coordination modes of V ions on catalytic activity, we chose a previously obtained structure **VMOP‐14** as the catalyst for a comparison test, which consists only of the octahedral 6‐coordinate {VO_6_} (Figure S16, Supporting Information). The results show that **VMOP‐14** has almost no catalytic activity for the oxidation of sulfides under the same reaction conditions. In situ, electron paramagnetic resonance (EPR) tests were performed to explore the reactive species by using dimethylpyridine N‐oxide (DMPO) as a spin trap (Figure S17, Supporting Information). No EPR signal could be detected in the absence of either catalyst. On V_66_‐MOP, a remarkable radical signal peak was observed at room temperature, which was ascribed to that of DMPO‐·OH. While only a very weak signal peak appeared when the catalyst was replaced with **VMOP‐14**.

To gain deeper insights into the catalytic performance of V‐MOPs, density functional theory (DFT) calculations are performed to elucidate the reaction mechanism. VOSO_4_ is used to explore the mechanism details of the oxidation process due to the similar reactivity and simplicity of calculation. As shown in Figure S18 (Supporting Information), multiple activation pathways have been considered. Firstly, VOSO_4_ (**Int1**) coordinates with H_2_O_2_, forming a stable intermediate **Int2**. Subsequently, the homolytic cleavage of H_2_O_2_ occurs via the transition state **TS1**, generating the intermediate **Int3**. The Gibbs reaction energy (*∆G°*) and Gibbs activation energy (*∆G°‡*) are −4.6 and 16.5 kcal mol^−1^, respectively. Next, after the dissociation of hydroxyl radical (•OH), the intermediate **Int4** interacts with the hydroxyl hydrogen connected to V through hydrogen atom transfer (HAT) process through transition state **TS2** and forms hydrated vanadium oxysulfate **Int5**, with a *∆G°‡* of 16.1 kcal mol^−1^. The •OH radical‐mediated dehydration process has more kinetic advantages compared to non‐radical‐mediated dehydration by transition state **TS2a** (16.1 vs 20.0 kcal mol^−1^). Another possible pathway is excluded due to the relatively high activation energy (39.4 kcal mol^−1^) of the transition state **TS2b**, in which the elementary step of HAT between oxygen atoms in H₂O₂ is considered. Next, through a ligand exchange process with sulfoxide and water, the sulfoxide‐coordinated intermediate **Int6** is generated. Finally, oxygen atom transfer occurs via the V‒O‒S transition state (**TS3**), facilitating the oxidation of sulfoxide to form the target product **Int7**. After the dissociation of sulfone, the entire catalytic cycle is completed. We found, in the proposed mechanism, that the rate‐determining step (RDS) is the homolytic activation of H_2_O_2_. Consequently, the essence that determines the catalytic performance is H_2_O_2_ activation capacity of catalysts, as suggested by the in situ EPR results. Frontier molecular orbitals were used to further analyze the catalytic activity of the two fragments {VV_5_O_11_} and {V_5_O_10_}, considering the periodic structure of V‐MOPs (Figure S19, Supporting Information). The results show that the energy levels of the two units are close to each other in the SOMO region, which corresponds to the similar catalytic activity of the two V‐MOPs **V_60_** and **V_66_**. The above observations and analyses then led us to a reasonable hypothesis that the tetragonal conical 5‐coordinated {VO_5_} units in the V clusters possess a better H_2_O_2_ activation capacity. We anticipate that the principle demonstrated here will be pivotal in designing highly efficient POVs catalysts for low‐temperature H_2_O_2_ activation for various oxidative reactions, although the influence of ligands is not investigated.

## 3 Conclusion

In conclusion, we have demonstrated a precise self‐assembly of fullerene‐like **V_60_‐MOP** constructed from the first discovered polyoxovanadates five‐membered ring **V*_5_*** units. In this spherical cage, twelve **V*_5_*** pentagonal molecular tiles were spliced with twenty planar rigid organic linkers BTC assembled to such a fascinating cluster with I*_h_* symmetry. In situ modifications were successfully used to insert {MO_5_} (M = V/Mo) in the center of the **V*_5_*** ring, resulting in two other isomorphic spherical cages **V_72_‐MOP** and **V_60_Mo_12_‐MOP**. A rare **V_66_‐MOP** assembled from two kinds of pentagonal molecular tiles was also obtained, where six {VV_5_} units are arrayed on the equatorial ring of the sphere while the other six **V*_5_*** units are distributed on the hemispherical surfaces on both sides of the equator, which results in a decrease in the symmetry of the spherical molecule due to the inhomogeneous arrangement of the two kinds of units. Furthermore, V‐MOPs can be regarded as a kind of catalyst for sulfide oxidation, showing obvious catalytic activity for a series of sulfide substrates converted to sulphoxide in greater quantities. The discovered **V*_5_*** unit not only embodied a crucial role in the construction of fascinating spherical polyhedral structures, but also confirmed that it also provides an important active site in catalytic reactions.

## Conflict of Interest

The authors declare no conflict of interest.

## Supporting information



Supporting Information

Supporting cif

## Data Availability

The data that support the findings of this study are available from the corresponding author upon reasonable request.
