# Supplementary material for: Self‐Assembly Engineering of Fullerene‐Like Polyhedra: V60, V66, V72 From {MV5} Pentagonal Second Building Block
Source: Adv Sci (Weinh). 2025 Mar 24;12(19):2408863. doi: 10.1002/advs.202408863 (PMC12097101; doi:10.1002/advs.202408863)
Supplement: Supplementary file 1 — Supporting Information [file ADVS-12-2408863-s001.pdf]

ADVANCED SCIENCE
Open Access

Supporting Information

for *Adv. Sci.*, DOI 10.1002/advs.202408863

Self-Assembly Engineering of Fullerene-Like Polyhedra: V_{60}, V_{66}, V_{72} From {MV_5} Pentagonal Second Building Block

Hongmei Gan, Linyan Bao, Na Xu, Baoshan Hou, Xinlong Wang and Zhongmin Su**

Supplementary Materials for

Self-assembly engineering of fullerene-like polyhedra: V_{60}, V_{66}, V_{72} from $\{MV_5\}$ pentagonal second building block

Hongmei Gan,[1,2] Linyan Bao,[1] Na Xu,[3] Baoshan Hou,[3] Xinlong Wang[3]* and Zhongmin Su[1]*

[1] State Key Laboratory of Supramolecular Structure and Materials. Jilin University. Changchun, Jilin, 130024 (China)

[2] Key Laboratory of Chemistry and Chemical Engineering on Heavy-Carbon Resources. Yili Normal University. Yining, Xinjiang, 835000 (China)

[3] Key Lab of Polyoxometalate Science of Ministry of Education. Northeast Normal University. Changchun, Jilin, 130024 (China)

*Corresponding Author(s): zmsu@nenu.edu.cn; wangxl824@nenu.edu.cn.

General Experimental Section

All the reagents were obtained from commercial sources and used without further purification. Powder X–ray diffraction (PXRD) measurement was recorded ranging from 5 to 50º at room temperature on a Siemens D5005 diffractometer with Cu-*Kα* (λ = 1.5418 Å). Thermogravimetric analysis (TGA) of the samples was performed using a Perkin–Elmer TG-7 analyzer heated from room temperature to 800 ºC under nitrogen at the heating rate of 10 °C·min^{-1}. IR spectrum was performed in the range 4000-500 cm^{-1} using KBr pellets on an Alpha Centaurt FT/IR spectrophotometer. X-ray photoelectron spectroscopy analyses were performed on a VG ESCALABMKII spectrometer with an Al-Kα (1486.6 eV) achromatic X-ray source. The vacuum inside the analysis chamber was maintained at 6.2×10^{-6} Pa during the analysis. Variable temperature magnetic susceptibility data were obtained in the temperature range of 2-300 K using a SQUID magnetometer (Quantum Design, MPMS-5) with an applied field of 1000 Oe.

Synthesis of V_{60}-MOP

The steam-thermal method: All the solids are added to a 3 ml quartz glass tube, while all liquids are directly poured into Teflon-lined stainless steel vessel. After that, the glass tube is placed in the liner to ensure that the liquid does not go over the tube.

$VOSO_4 \cdot 5H_2O$ (0.02 g), benzene-1,3,5-tricaboxyllic acid (H_3BTC, 0.015 g) and Na_3VO_4 (0.01 g). 2 mL DMA (N, N-Dimethylacetamide), 0.5 mL MeCN (acetonitrile) were transferred to a Teflon-lined stainless steel vessel heated to 130 °C and held at this temperature for 24h. After slow cooling to room temperature, bright green octahedral crystals were obtained (washed with MeOH) with a yield of 37 % based on H_3BTC. $C_{212}H_{188}N_{16}O_{308}S_{14}V_{72}$: C, 21.21; H, 1.58; N, 1.87. Found: C, 22.37; H, 1.71; N, 1.68. IR (KBr, cm^{-1}): 3432 (s), 2931 (w), 2793 (w), 2461 (w), 1654 (s), 1618 (s), 1562 (m),

1439 (s), 1383 (s), 1256 (w), 1138 (s), 1103 (s), 975 (s), 801 (w), 765 (s), 720 (s), 663 (m), 622 (m), 597 (w).

Synthesis of V_{72}-MOP

$VOSO_4 \cdot 5H_2O$ (0.025 g), H_3BTC (0.02 g) and Na_3VO_4 (0.01 g) in 2 mL DMF (N, N-dimethylformamide), 0.5 mL MeCN (acetonitrile) were transferred to a Teflon-lined stainless steel vessel heated to 130 ºC and held at this temperature for 48h. After slow cooling to room temperature, bright green block crystals were obtained (washed with MeOH) with a yield of 56 % based on H_3BTC. Elemental analysis for $C_{180}H_{60}O_{300}S_{12}V_{72}$: C, 19.83; H, 0.76; N, 1.48. Found: C, 19.16; H, 0.55; N, 1.23. FTIR (KBr, cm^{-1}): 3078 (m), 2772(m), 1715(w), 1654(w), 1617(vs), 1563(s), 1446(s), 1387(vs), 1136(s), 983(s), 759(w), 720(s), 622(m), 498(m).

Synthesis of $V_{60}Mo_{12}$-MOP

$VOSO_4 \cdot 5H_2O$(0.025g), Na3VO4 (0.02 g), $(NH_4)_6Mo_7O_{24}$,$4H_2O$ (0.01g) and H_3BTC(0.015g) in 2 mL DMF (N, N-dimethylformamide), 0.5 mL MeCN (acetonitrile) were transferred to a Teflon-lined stainless steel vessel heated to 150 ºC and held at this temperature for 48h. After slow cooling to room temperature, green strip crystals were obtained (washed with MeOH) with a yield of 43 % based on H_3BTC. Elemental analysis for C_{180} Mo_{12} O_{252} V_{60} H_{60}: C, 21.05; H, 0.76; N, 1.58. Found: C, 21.83; H, 0.56; N, 1.24. FTIR (KBr, cm-1): 3037 (m), 1654(w), 1615(vs), 1561(s), 1444(s), 1388(vs), 1104(s), 984(s), 806(w), 762(w), 715(s), 616(s), 572(w), 504(m).

Synthesis of V_{66}-MOP

$VOSO_4 \cdot xH_2O$ (0.025 g), benzene-1,3,5-tricaboxyllic acid (0.02 g) and Na_3VO_4 (0.015 g) in 2 mL DMF (N, N-dimethylformamide), 0.5 mL MeCN (acetonitrile) and 2 drops of HBr were transferred to a Teflon-lined stainless steel vessel heated to 160 ºC and held at this temperature for 48h. After slow cooling to room temperature, dark brown block crystals were obtained (washed with MeOH) with a yield of 82% based on H_3BTC. C_{180} H_{60} O_{291} S_{12} V_{66}: C, 21.21; H, 1.58; N, 1.87. Found: C, 22.37; H, 1.71; N, 1.68. FTIR (KBr, cm^{-1}):3428 (m), 3080(m), 1615(w), 1558(vs), 1445(s), 1388(vs), 1106(m), 977(m), 837(w), 760(s), 719(vs), 614(w), 572(w), 503(m).

Crystal structure determination and refinements

The crystallographic data of V-MOPs are given in Table S1. Intensity data collections were performed on a Bruker D8–Venture diffractometer with a Turbo X-ray Source (Cu Kα radiation, λ = 1.5418 Å). Absorption corrections were applied using a multi–scan technique. The data frames were collected using the program APEX 3 and processed using the program SAINT routine in APEX 3. The structures were solved by direct methods and refined by the full matrix least-squares on F^2 using the SHELXL–2014 program. The diffused electron densities resulting from these residual solvent molecules were removed from the data set using the SQUEEZE routine of PLATON and refined further using the data generated. The restrained DFIX, SIMU, ISOR instructions were used to make the structures more reasonable. The formula unit was obtained through a combination of elemental analyses and thermogravimetric characterization. CCDC number of 2370848 for **V_{60}-MOP**,2370849 for **V_{72}-MOP**,2370850 for **$V_{60}Mo_{12}$-MOP**, 2370851 for **V_{66}-MOP**.

Results and discussion

Table S1. The Crystallographic data for **V-MOPs**.

Identification code	**V_{60}-MOP**	**V_{72}-MOP**	**$V_{60}Mo_{12}$-MOP**	**V_{66}-MOP**
Empirical formula	C_{180} H_{60} O_{288} S_{12} V_{60}	C_{180} H_{60} $O_{300}S_{12}$ V_{72}	C_{180} Mo_{12} O_{252} V_{60}	C_{180} H_{60} O_{306} S_{15} V_{66}
Formula weight	10270.58	11266.80	10457.49	10961.00
Temperature/K	298.0	298.0	173(2)	100.15
Crystal system	Cubic	Trigonal	Monoclinic	Hexagonal
Space group	*Fm*—3	*R*—3*m*	*I*2/*m*	*P*6$_3$*mc*
a/Å	42.5524(10)	29.8478(12)	30.067(8)	29.6130(8)
b/Å	42.5524(10)	29.8478(12)	29.792(8)	29.6130(8)
c/Å	42.5524(10)	79.521(8)	48.123(13)	52.307(2)
α/°	90	90	90	90
β/°	90	90	100.685(15)	90
γ/°	90	120	90	120
Volume/Å^3	77050(5)	61353(8)	42359(20)	39724(3)
Z	4	2	2	2
ρcalcg/cm^3	0.885	0.863	0.820	0.916
μ/mm1	6.611	7.209	7.089	7.073
F(000)	20064	15409	10016	10692
Radiation	CuKα (λ = 1.54178)	CuKα (λ = 1.54178)	CuKα (λ = 1.54178)	CuKα (λ = 1.54178)
2θ range/°	2.937 to 40.021	2.039 to 33.525	2.106 to 31.410	3.836 to 127.864
Reflections collected	19211	15710	22496	275797
Independent reflections	2104 [Rint= 0.0564, Rsigma=0.0338]	2887 [Rint= 0.0755, Rsigma=0.0514]	6902 [Rint = 0.1453, Rsigma = 0.1201]	23258 [Rint = 0.0781, Rsigma =0.0521]
Goodness-of-fit on F_2	1.874	1.049	1.483	1.034
Final R indexes [I > = 2σ (I)]	R_1 = 0.1081, wR_2 = 0.3583	R_1 =0.0868, wR_2 = 0.2386	R_1 = 0.1178, wR_2 = 0.3166	R_1 = 0.0618, wR_2 = 0.1790

Final R indexes [all data]	R_1 = 0.1206, wR_2 = 0.3789	R_1 = 0.0984, wR_2 = 0.2492	R_1 = 0.1489, wR_2 = 0.3423	R_1 = 0.0787, wR_2 = 0.2079

[a]$R_1 = \Sigma||F_o|-|F_c||/\Sigma|F_o|$; [b]$wR_2=\{\Sigma [w(F_o^2-F_c^2)^2]/\Sigma [w(F_o^2)^2]\}^{1/2}$

Table S2. BVS results for the vanadium ions in **V_{60}**.

**V_{60}-MOP**		Bond distance (Å)	BVS calc. for V (III)	BVS calc. for V (IV)	BVS calc. for V (V)
**V1**	O(1)	2.073(15)	0.417	0.453	0.482
	O(6)	1.593(12)	1.524	1.658	1.764
	O(7)	2.028(15)	0.470	0.512	0.544
	O(10)	1.999(12)	0.509	0.553	0.589
	O(36)	1.957(13)	0.570	0.620	0.660
	O(45)	2.181(14)	0.311	0.338	0.360
			3.801	**4.134**	4.399
**V2**	O(9)	1.582(13)	1.570	1.708	1.817
	O(10)#2	1.955(12)	0.573	0.623	0.663
	O(25)#2	2.013(15)	0.490	0.533	0.567
	O(38)	1.966(11)	0.556	0.605	0.644
	O(43)	2.416(14)	0.165	0.179	0.191
	O(3)#1	2.027(12)	0.472	0.513	0.546
			3.826	**4.161**	4.428
**V3**	O(5)#2	2.018(16)	0.483	0.526	0.559
	O(5)	2.018(16)	0.483	0.526	0.559
	O(14)	1.575(18)	1.600	1.740	1.852
	O(36)#2	1.963(13)	0.561	0.610	0.649
	O(36)	1.963(13)	0.561	0.610	0.649
			3.689	**4.011**	4.268

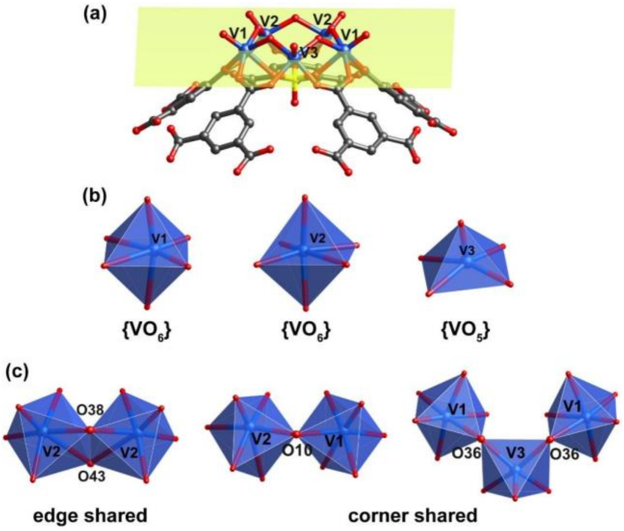

Figure S1. (a) Ball–and–stick views of $\{V_5S\}$ cluster in **V_{60}-MOP**. **(b)** Coordination mode of vanadium atoms in **V_5** cluster. **(c)** Connection mode of edge shared and corner shared in **V_5** cluster. Color codes: $\{VO_6\}/\{VO_5\}$ polyhedron, blue; V, blue; S, yellow; O, red; C, gray. For clarity, hydrogen atoms are omitted.

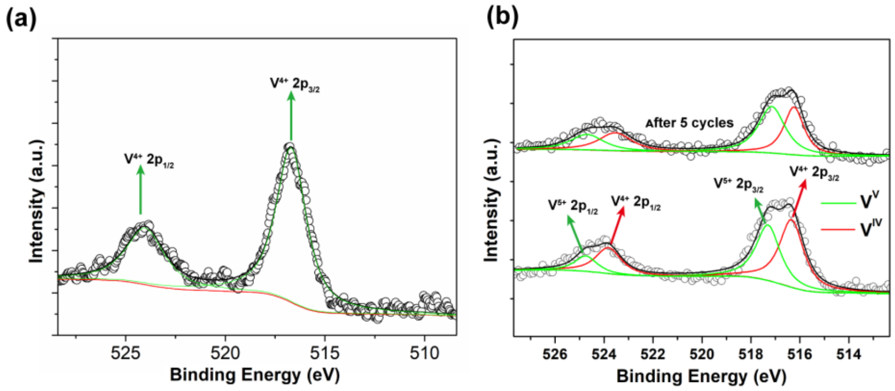

Figure S2. X–ray photoelectron spectroscopy of **(a) V_{60}-MOP** and **(b) V_{66}-MOP**.

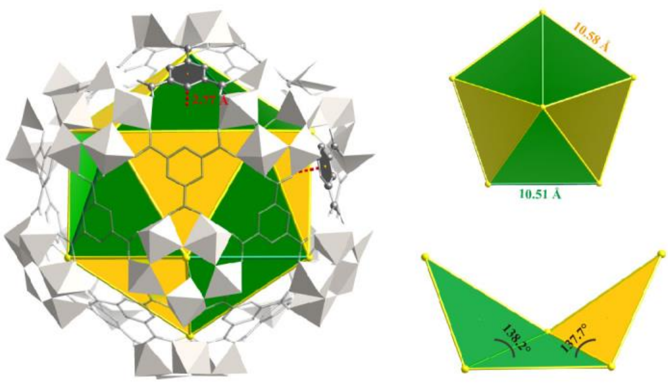

Figure S3. Icosahedron contains two kinds of edges (10.51 green, 10.58 yellow), and two kinds of dihedral angle.

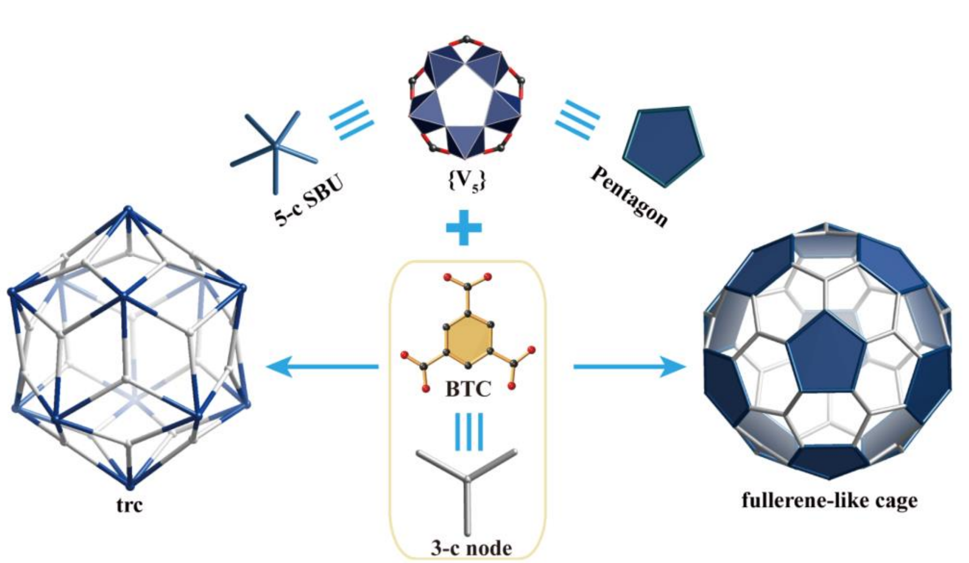

Figure S4. The simplest topology of **V_{60}-MOP.**

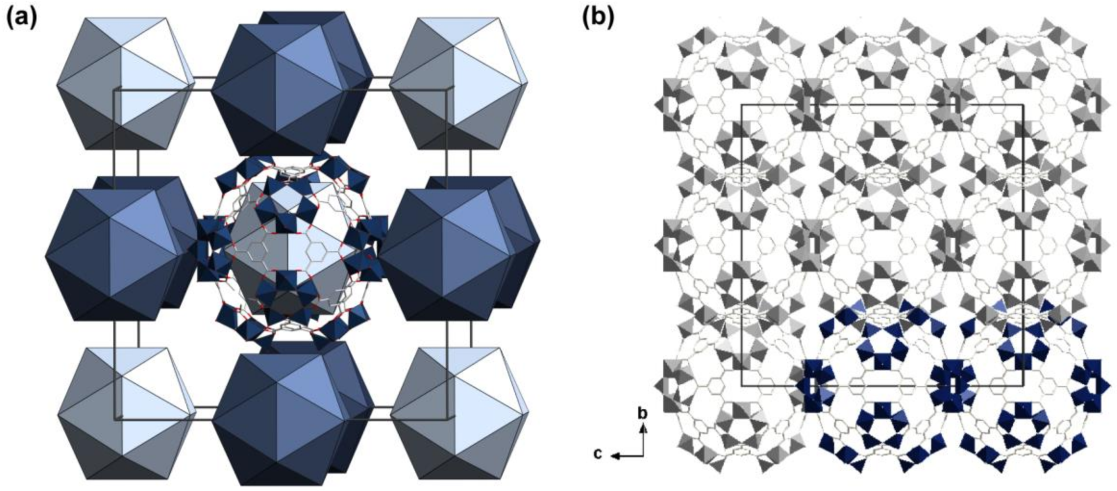

Figure S5. The cubic packing arrangements of **V_{60}-MOP** with view in the direction of the crystallographic *a* axis.

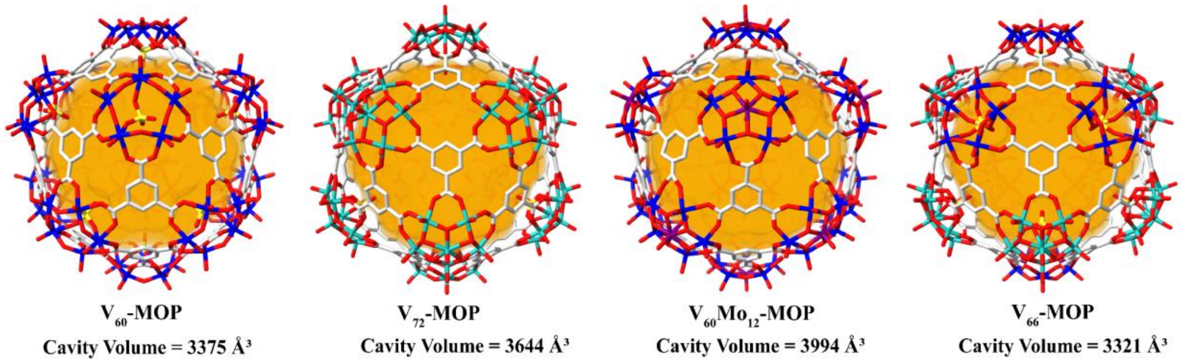

Figure S6. VOIDOO-calculated void spaces as shown (yellow mesh) within the crystal structures of **V-MOPs**.

Table S3. The BVS calculations for **V_{66}**-MOP

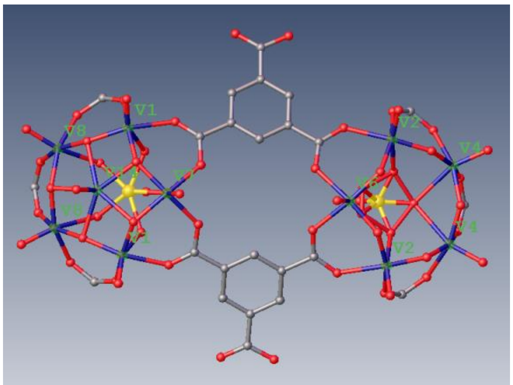

Atom	V6	V2	V4	V7	V1	V8	V14
BVS calc. for V	4.15	4.15	4.18	4.09	4.28	4.23	5.03

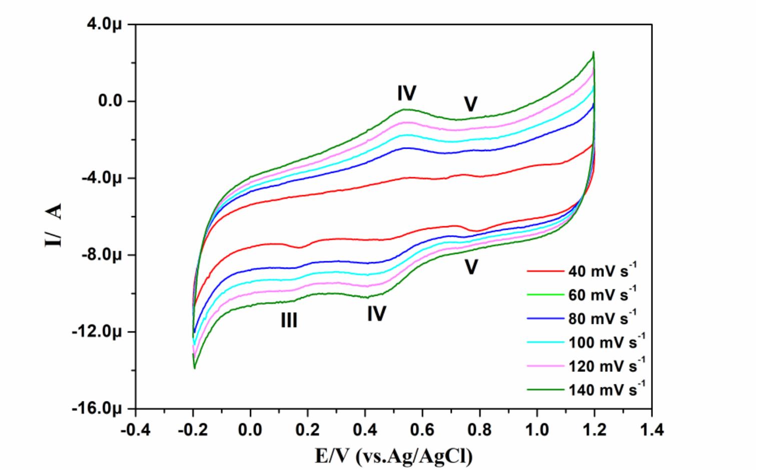

Figure S7. CV curves of $\mathbf{V_{66}}$**-MOP** in 0.5 M H_2SO_4 with different scan rates.

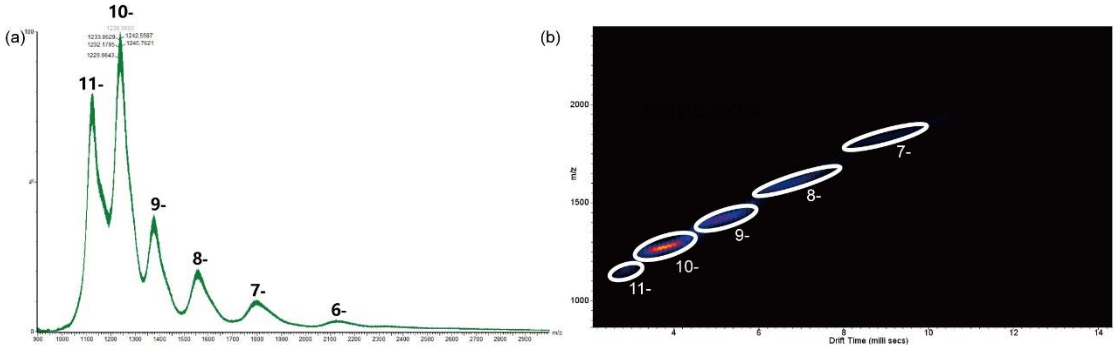

Figure S8. (a) ESI-MS and (b) TWIM-MS plots (m/z vs drift time) of $\mathbf{V_{66}}$**-MOP**.

Electrospray ionization MS (ESI-MS) of V_{66} shows a dominant set of peaks with continuous charge states ranging from 6- to 11- due to the successive loss of the counterion (Figure S8a). The average molar mass of V_{66} is deduced to be 13 KDa, matching well with the molecular formula of $[C_{260}H_{380}V_{66}N_{40}O_{306}S_{15}]$. Traveling wave ion mobility MS (TWIM-MS) of V_{66} shows a series of bands with a narrow drift time at each charge state, indicating that no other isomers or structural

conformers exist (Figure S8b).

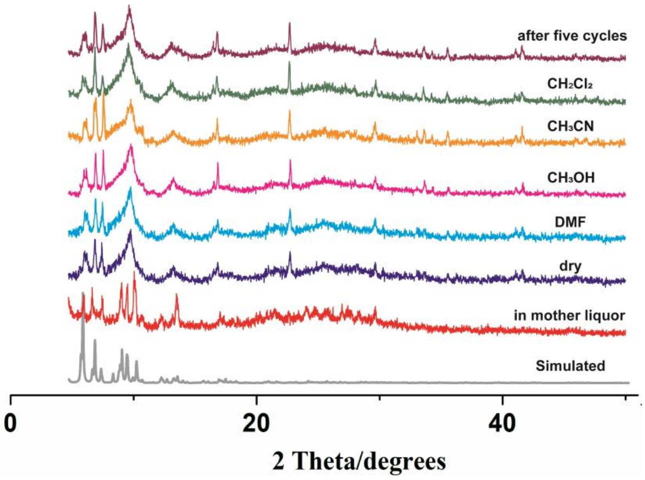

Figure S9. PXRD in different solvents and after five cycles for **V_{66}-MOP**

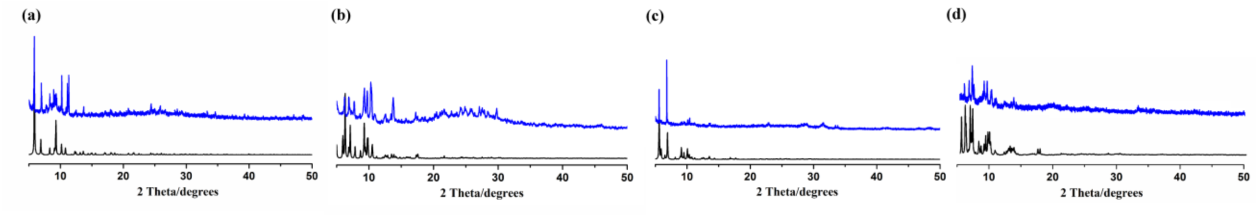

Figure S10. Experimental and simulated powder X–Ray diffraction patterns (PXRD) for **V_{60}-MOP** (a); **V_{66}-MOP** (b); **V_{72}-MOP** (c); **$V_{60}Mo_{12}$-MOP** (d).

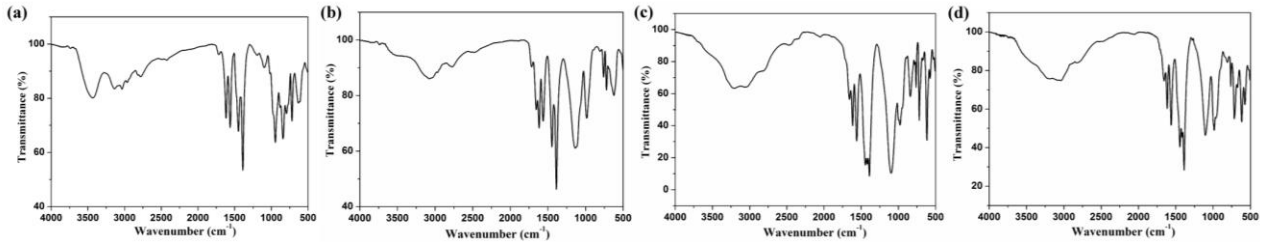

Figure S11. Infrared spectroscopy for **V_{60}-MOP** (a); **V_{66}-MOP** (b); **V_{72}-MOP** (c); **$V_{60}Mo_{12}$-MOP** (d).

As for the IR spectrum，500-1200cm^{-1} region contains the absorption band of metal-oxygen tensile vibration, which usually corresponds to the characteristic region of polyoxometalates. The two the characteristic peaks at about 715-763cm^{-1} are attributed to the V-O-V bridging segments. The peaks around 970-1138cm^{-1} are associated with SO_4^{2-} in the structures. The characteristic peaks at 1560-1600cm^{-1} are due to the carboxylic acid in BTC ligands, while the peaks near about 800cm^{-1} and 2900cm^{-1} are related to the C-H bond of the benzene ring. Finally, the peaks band at 3000-3500 cm^{-1} indicates the presence of $[(CH_3)_2NH_2]^+$ cations in these structures.

Table S4. Functional group identification in the IR for V-MOPs

	V_{60}	V_{66}	V_{72}	$V_{60}Mo_{12}$
V-O-V	719，763	720，760	719，759	717，761
V=O	944	977	979	985
SO_4^{2-}	1097	1103	1133	1099
C=O	1386，1619	1386，1618	1394，1619	1384，1618
C=C	1444，1564	1446，1556	1448，1560	1448，1558
C-H	3035	3037	3081	3031

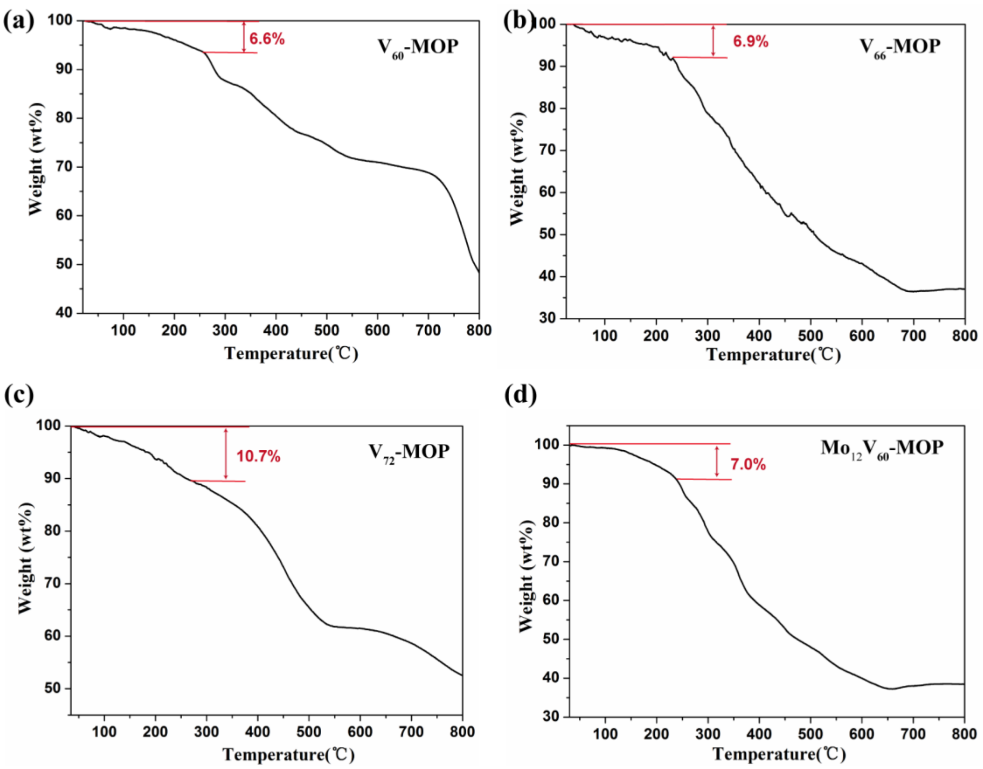

Figure S12. The TG curves for **V_{60}-MOP** (a); **V_{66}-MOP** (b); **V_{72}-MOP** (c); **$V_{60}Mo_{12}$-MOP** (d).

Thermogravimetric analysis (TGA) in N_2 of V-MOPs exhibit similar continuous weight loss process. The first weight loss range from 25 °C to 230-258 °C attribute to the lost of solvent molecules (MeOH+DMF or DMA). V-MOPs can maintain good thermal stability at least until 200 °C, while the skeleton will collapse as the temperature continues to rise.

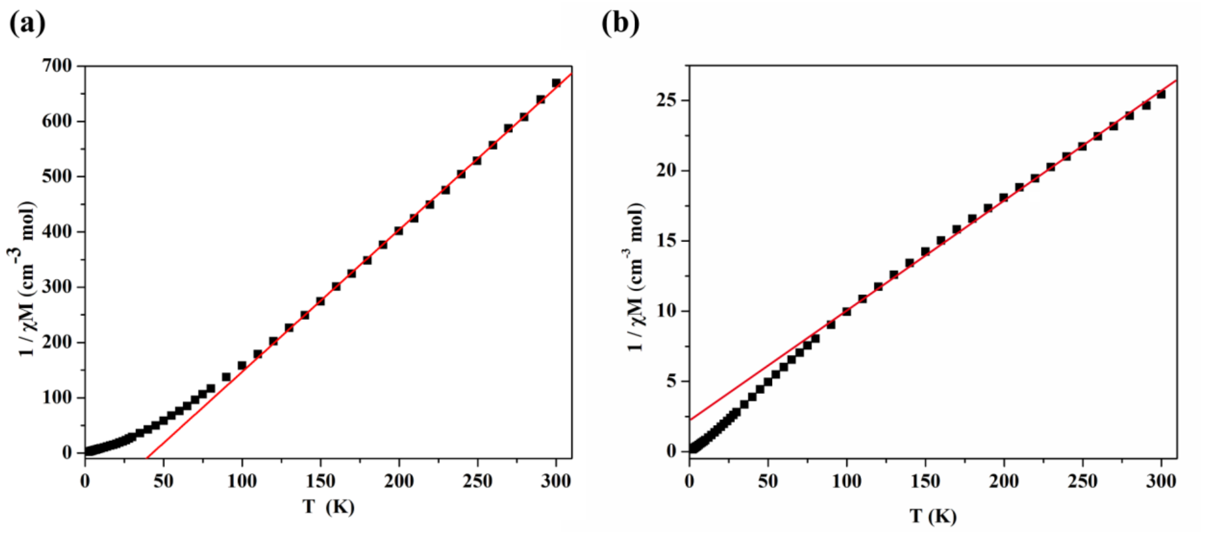

Figure S13. The temperature dependence of the inverse magnetic susceptibility χ_M^{-1} for **V_{60}-MOP (a)** and **V_{66}-MOP (b)** between 2 and 300 K. The solid red line was generated from the best fit by the Curie-Weiss expression in the range of 100–300 K.

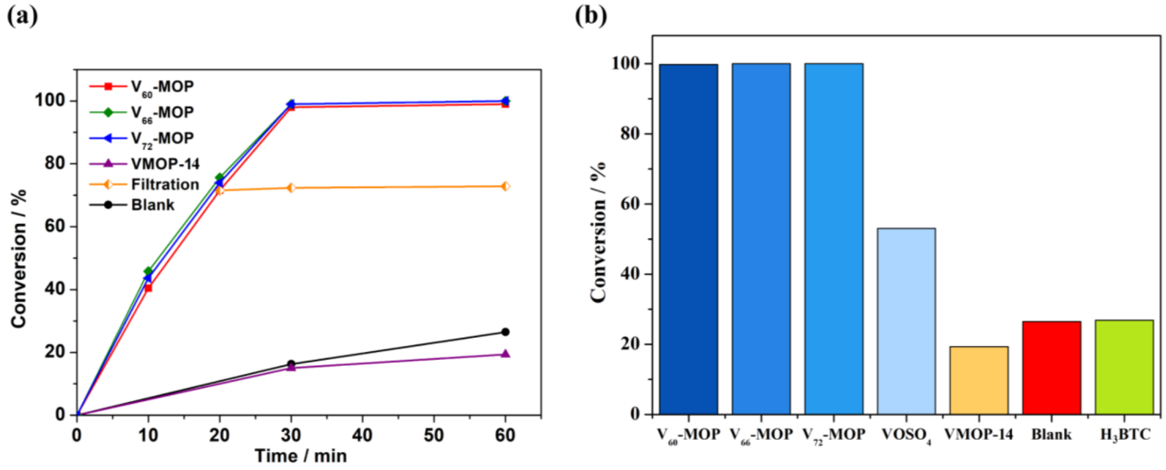

Figure S14. (a) Kinetics of the methyl phenyl sulfide sulfoxidation reaction catalyzed by V-MOPs; (b) Control experiments of sulfide oxidation. Reaction conditions: sulfide (0.4 mmol), V-MOP (0.002 mmol), H_2O_2 (1mmol) and MeOH (5 mL), 25 °C, 1 h. blank means no catalyst was added.

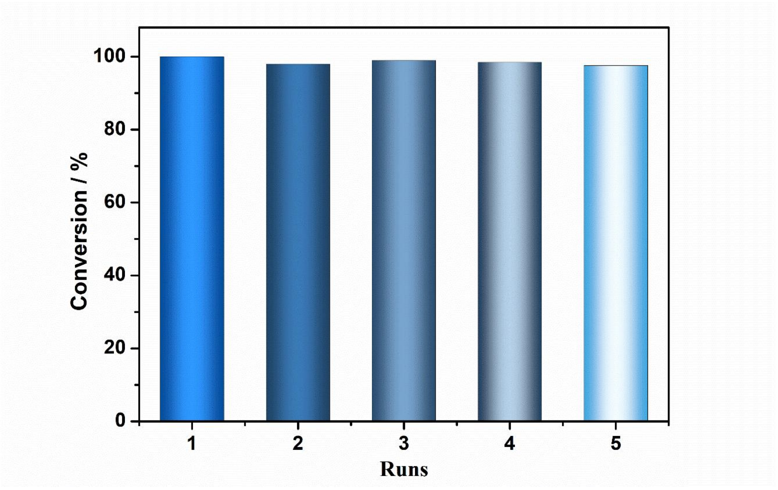

Figure S15. Circular experimental diagram of MBT catalyzed by **V_{66}-MOP**.

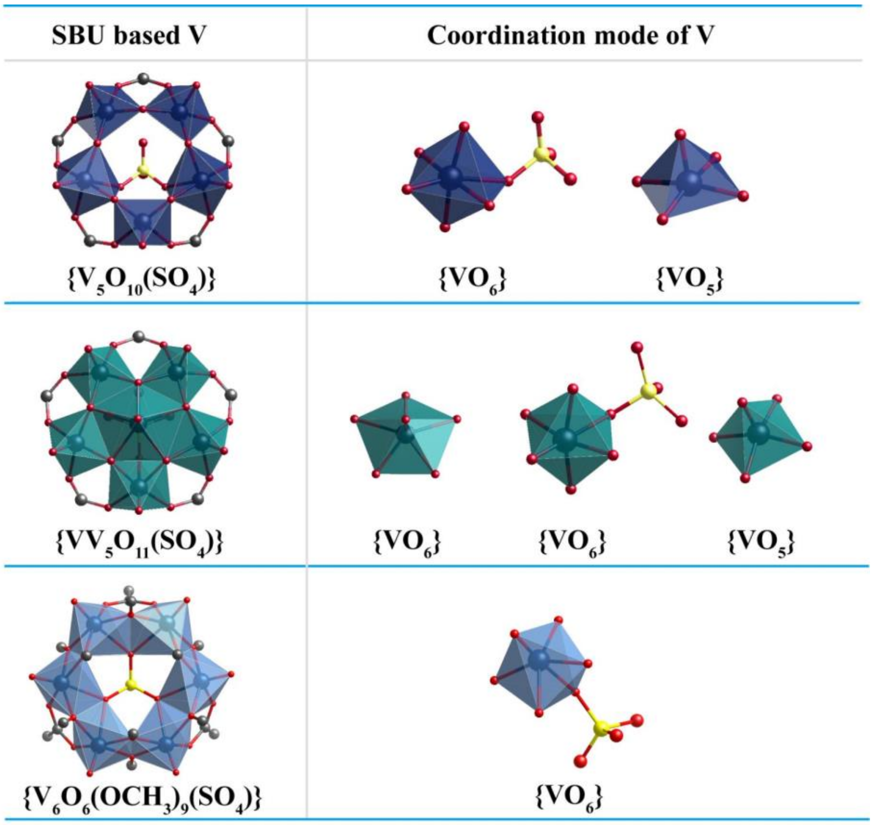

Figure S16. Coordination mode of vanadium atoms in {V_5S}, {VV_5S} and {V_6S} SBUs from **V_{66}-MOP** and **VMOP-14** (light blue).

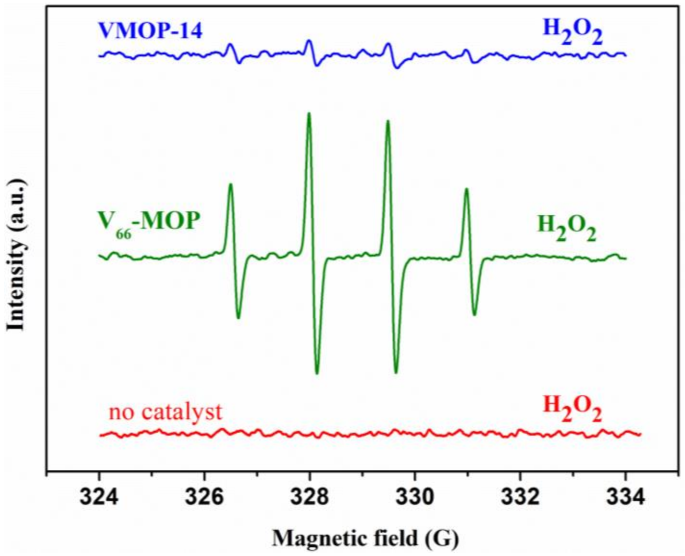

Figure S17. EPR spectra of the spin-trapping experiments with DMPO as spin-trapping agent.

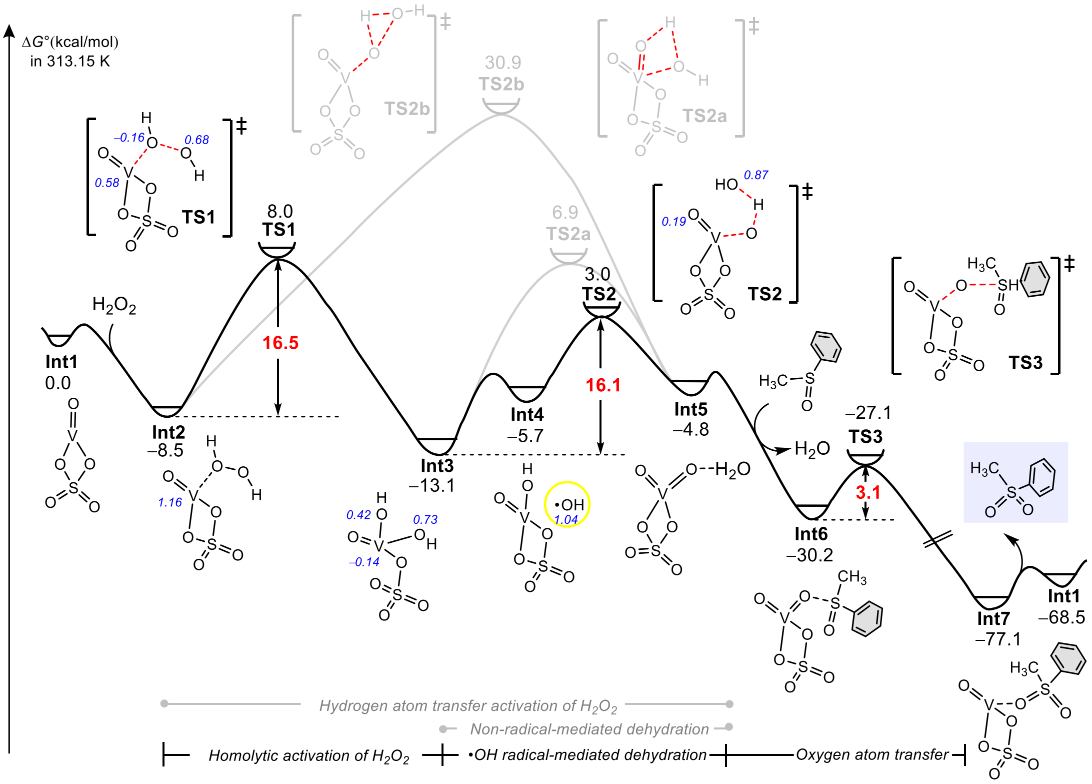

Figure S18. Gibbs free energy profile (in kcal/mol) of the sulfoxide oxidized by vanadium oxysulfate. The red bold font is the Gibbs activation energy of the corresponding elementary step. The blue font represents the spin density value of the corresponding atom.

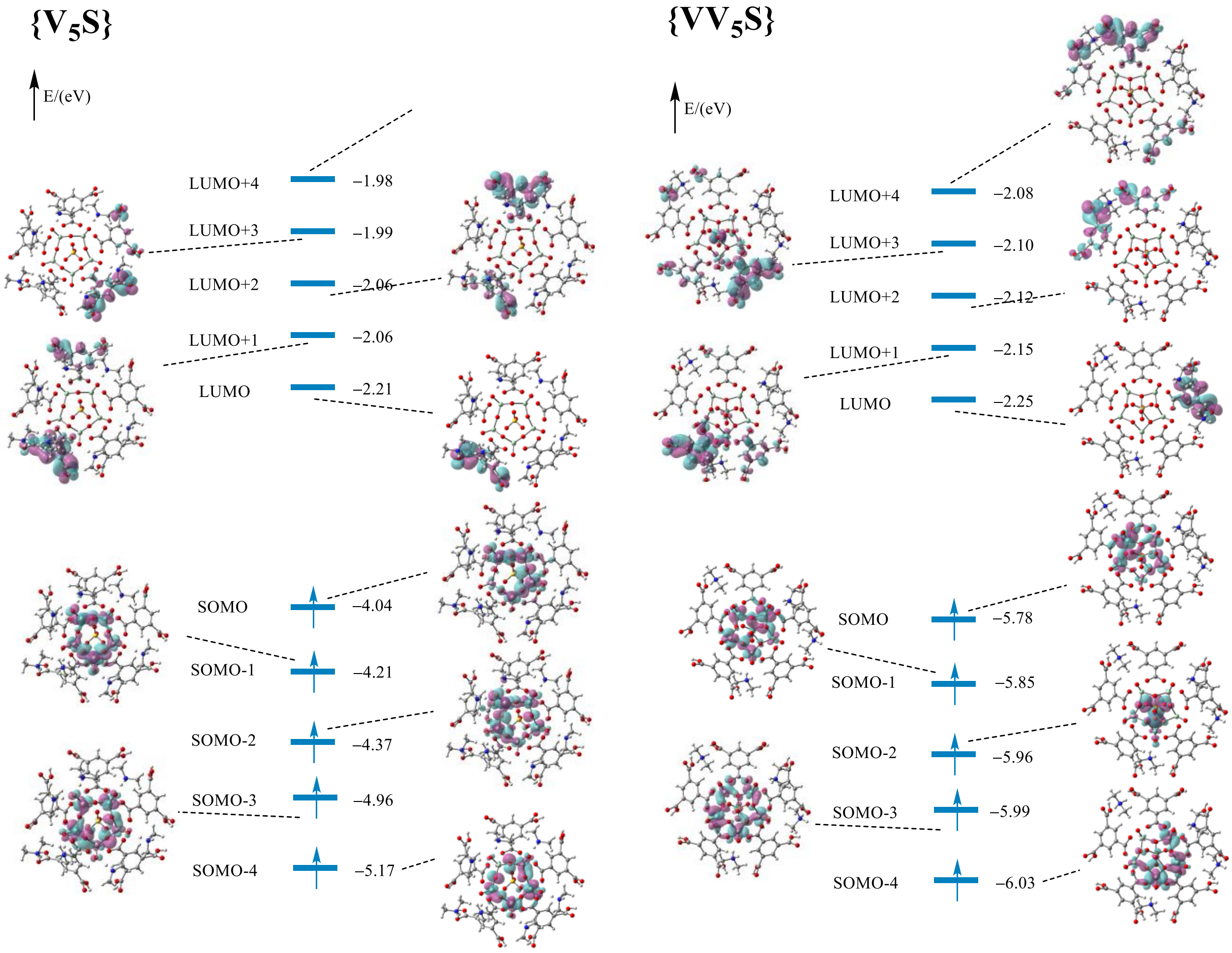

Figure S19. Frontier molecular orbital analysis of two cluster fragments.
